# Clinical application of autologous technetium-99m-labelled eosinophils to detect focal eosinophilic inflammation in the lung

**DOI:** 10.1136/thoraxjnl-2015-207156

**Published:** 2015-06-24

**Authors:** Chrystalla Loutsios, Neda Farahi, Rosalind Simmonds, Ian Cullum, Daniel Gillett, Chandra Solanki, Kishor Solanki, John Buscombe, Alison M Condliffe, A Mike Peters, Edwin R Chilvers

**Affiliations:** 1 Division of Respiratory Medicine, Department of Medicine, University of Cambridge, Cambridge, UK; 2 Department of Nuclear Medicine, Addenbrooke's Hospital, Cambridge, UK; 3 Clinical Imaging Sciences Centre, Brighton and Sussex Medical School, Brighton, UK

**Keywords:** Eosinophil Biology, Imaging/CT MRI etc, Pulmonary eosinophilia

## Abstract

The detection of focal eosinophilic inflammation by non-invasive means may aid the diagnosis and follow-up of a variety of pulmonary pathologies. All current methods of detection involve invasive sampling, which may be contraindicated or too high-risk to be performed safely. The use of injected autologous technetium-99m (Tc-99m)-labelled eosinophils coupled to single-photon emission computed tomography (SPECT) has been demonstrated to localise eosinophilic inflammation in the lungs of a patient with antineutrophil cytoplasmic antibody-positive vasculitis. Here, we report on the utility of this technique to detect active eosinophilic inflammation in a patient with focal lung inflammation where a biopsy was contraindicated.

**To the editor**

Our patient, a 69-year old with a background of asthma, presented with a 14-month history of increasing wheeze and cough, followed by acute deterioration with breathlessness, weight loss and anorexia. On admission, he was haemodynamically unstable and severely hypoxaemic. Thoracic computed tomography (CT) revealed a right-sided pneumothorax with peripheral ground glass opacities and consolidation throughout the left lung, raising the possibility of chronic eosinophilic pneumonia. Tests revealed marked blood eosinophilia of 2.3×10^9^/L and an elevated immunoglobulin E (IgE) at 910 kU/L; his rheumatoid factor, antinuclear antibody and antineutrophil cytoplasmic antibody were negative. His IgG_4_ was elevated at 6.54 g/L (normal range (NR) 0–1.3 g/L), suggestive of possible IgG_4_-related disease. His pneumothorax was successfully aspirated and he was given oxygen and empirical broad-spectrum antibiotics. As he was initially too unwell to undergo bronchoscopy, a Tc-99m-eosinophil-single-photon emission computed tomography (SPECT/CT) scan was undertaken, as this novel technique has been demonstrated previously to localise biopsy-proven pulmonary eosinophilic inflammation.^1^

Tc-99m-labelled autologous eosinophils were prepared using anti-CD16 microbeads as described previously.^2^ Tc-99m-eosinophils (192 MBq) were injected and dynamic gamma camera imaging commenced, followed by SPECT; a single low-dose CT to enable anatomical alignment was performed. Within 5 min, uptake of labelled cells was visible on planar images of his left lung (see figure 1A) in addition to the expected physiological uptake in the liver, spleen and bone marrow. Increased activity was further demonstrated in his left lung on cross-sectional SPECT (see figure 1B). Coronal, sagittal and transaxial SPECT/CT images indicated focal uptake of radiolabelled eosinophils in regions of the lung corresponding to abnormal areas on CT (see figure 1C–E). These results provided evidence of pulmonary eosinophilic inflammation and supported the early use of high-dose oral corticosteroids, which led to rapid clinical improvement. The patient's serum on the day of the SPECT was analysed using a multiplex cytokine and chemokine array (MSD MULTI-SPOT Array System) and showed raised levels (pg/mL) of interleukin (IL)-5, 2.2 (NR <0.62 pg/mL), IL-15, 5.6 (NR <3.01 pg/mL); eotaxin, 428 (NR <145 pg/mL), eotaxin 3, 130 (NR <8.73 pg/mL) and monocyte chemotactic protein-4 (MCP-4), 358 (NR <117 pg/mL).

**Figure 1:**
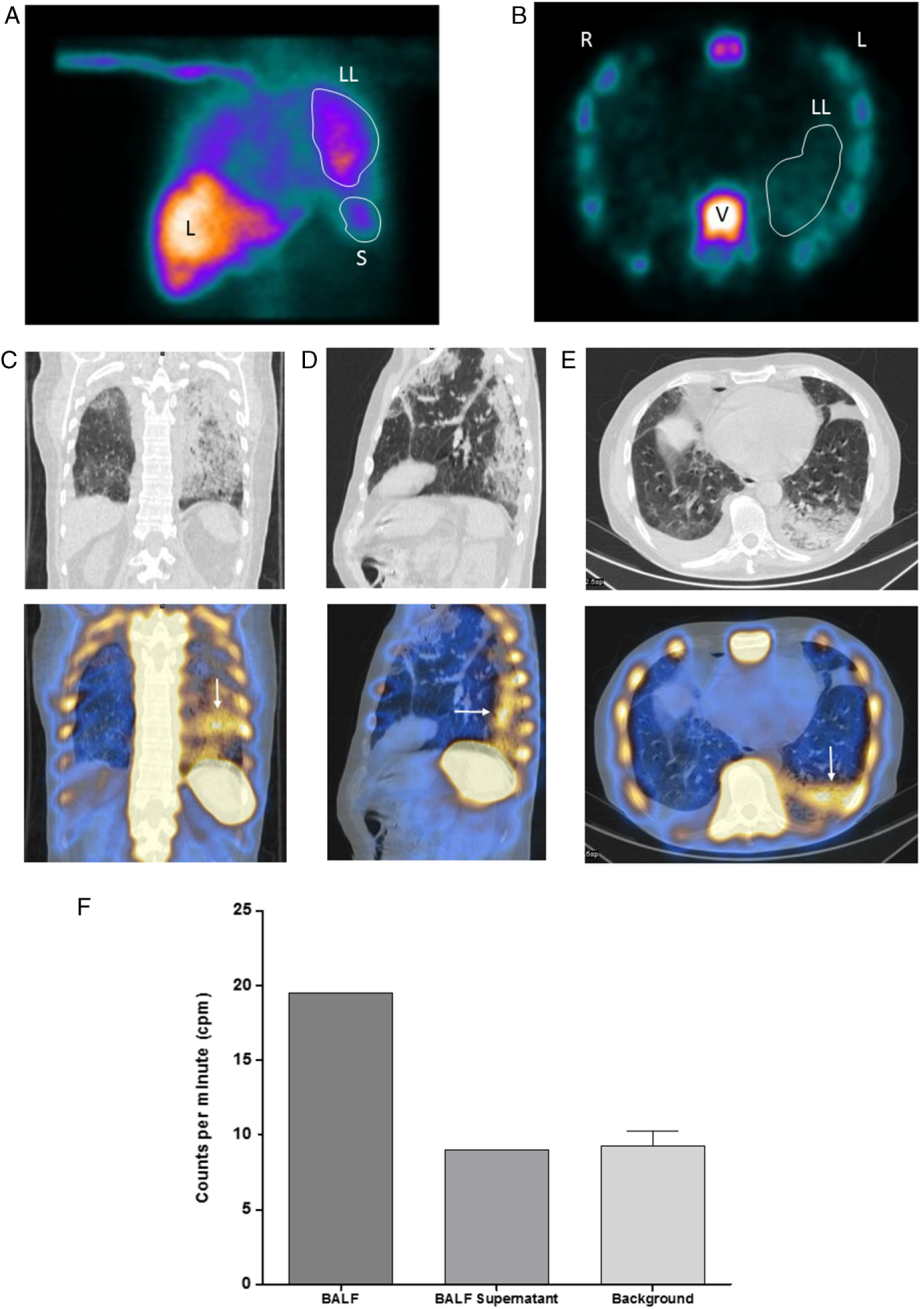
(A) Reframed composite image of the first 5 min of the dynamic planar gamma camera scan. Increased 99m-Tc-eosinophil uptake is visible in the left lung. LL, left lung; L, liver; S, spleen. (B) Transaxial single-photon emission computed tomography (SPECT) image showing increased activity in the vertebra and left lung (LL). L, left; R, right; V, vertebra. (C–E) Coronal, sagittal and transaxial sections through the lung. SPECT/CT (lower panel) demonstrating focal 99m-Tc-eosinophil uptake in areas of abnormality in the CT (upper panel), (white arrows). (F) Raw gamma counter results showing increased counts in the bronchoalveolar lavage fluid (BALF) compared with the BAL supernatant and natural background counts.

Within 48 h, his oxygenation had improved, allowing bronchoscopy. The bronchoalveolar lavage fluid (BALF) obtained had double the radioactive counts per minute (cpm) (19.5 cpm/mL) than the control fluid (phosphate-buffered saline) (9.3 cpm/mL) (see figure 1F). Counts in the supernatant (9 cpm/mL) after centrifugation were identical to the control, demonstrating that the radioactivity detected in the BALF was cell associated.

The patient made a rapid and complete recovery following treatment with oral prednisolone.

This case demonstrates the ability of Tc-99m-eosinophil-SPECT/CT to confirm pulmonary eosinophilic inflammation in patients where acquisition of BALF or lung biopsy was contraindicated. This methodology has the potential to be used clinically alongside or as a non-invasive alternative to conventional methods of detecting parenchymal eosinophilia. It may also aid in the translation of novel therapeutics by providing a platform to test their efficacy in reducing tissue eosinophilia.
